# ‘*Candidatus* Phytoplasma stylosanthis’, a novel taxon with a diverse host range in Australia, characterised using multilocus sequence analysis of 16S rRNA, *secA*, *tuf*, and *rp* genes

**DOI:** 10.1099/ijsem.0.004589

**Published:** 2020-12-08

**Authors:** Bianca Rodrigues Jardim, Wycliff M. Kinoti, Lucy T. T. Tran-Nguyen, Cherie Gambley, Brendan Rodoni, Fiona E. Constable

**Affiliations:** ^1^​ School of Applied Systems Biology, La Trobe University, Bundoora, Victoria, Australia; ^2^​ Agriculture Victoria Research, Department of Jobs, Precincts and Regions, AgriBio, Bundoora, Australia; ^3^​ Biosecurity and Animal Welfare, Department of Industry, Tourism and Trade, Darwin, Australia; ^4^​ Horticulture and Forestry Science, Department of Agriculture and Fisheries Maroochy Research Facility, Nambour, Australia

**Keywords:** phytopathogen, Mollicutes, *Stylosanthes *little leaf phytoplasma

## Abstract

In Australia, *Stylosanthes* little leaf (StLL) phytoplasma has been detected in *Stylosanthes scabra* Vogel, *Arachis pintoi* Krapov, *Saccharum officinarum* L., *Carica papaya* L., *Medicago sativa* L., and *Solanum tuberosum* L. The 16S rRNA gene sequence of StLL phytoplasma strains from *S. scabra*, *C. papaya, S. officinarum* and *S. tuberosum* were compared and share 99.93–100 % nucleotide sequence identity. Phylogenetic comparisons between the 16S rRNA genes of StLL phytoplasma and other ‘*Candidatus* Phytoplasma’ species indicate that StLL represents a distinct phytoplasma lineage. It shares its most recent known ancestry with ‘*Ca*. Phytoplasma luffae’ (16SrVIII-A), with which it has 97.17–97.25 % nucleotide identity. *In silico* RFLP analysis of the 16S rRNA amplicon using *i*PhyClassifier indicate that StLL phytoplasmas have a unique pattern (similarity coefficient below 0.85) that is most similar to that of *‘Ca*. Phytoplasma luffae’. The unique *in silico* RFLP patterns were confirmed *in vitro*. Nucleotide sequences of genes that are more variable than the 16S rRNA gene, namely *tuf* (tu-elongation factor), *secA* (partial translocation gene), and the partial *ribosomal protein* (*rp*) gene operon (*rps19-rpl22-rps3*), produced phylogenetic trees with similar branching patterns to the 16S rRNA gene tree. Sequence comparisons between the StLL 16S rRNA spacer region confirmed previous reports of *rrn* interoperon sequence heterogeneity for StLL, where the spacer region of *rrnB* encodes a complete tRNA-Isoleucine gene and the *rrnA spacer* region does not. Together these results suggest that the Australian phytoplasma, StLL, is unique according to the International Organization for Mycoplasmology (IRPCM) recommendations. The novel taxon ‘*Ca*. Phytoplasma stylosanthis’ is proposed, with the most recent strain from a potato crop in Victoria, Australia, serving as the reference strain (deposited in the Victorian Plant Pathology Herbarium as VPRI 43683).

## Introduction

Legume little leaf is a disease of various *Fabaceae* species, including the pasture legume *Stylosanthes,* and has been known in Australia since the 1950’s [1]. Infected plants show yellowing, bunching and proliferation, reduced leaf size and reduced survival [1–3]. In 1999, in Queensland, Australia, several phytoplasmas were detected for the first time in *Stylosanthes* species showing little leaf and witches’ broom symptoms [4]. In *Stylosanthes scabra*, a unique phytoplasma was detected in a mixed infection with three 16SrII-related phytoplasma strains sweet potato little leaf vinca 4 (SPLL-V4), pigeon pea little leaf (PLL) and tomato big bud (TBB). The unique phytoplasma was called *Stylosanthes* little leaf (StLL). In the same study, StLL was found in a mixed infection with SPLL-V4 and PLL in the *Fabaceae* species *Arachis pintoi* Krapov. and W.C. Gregory (Pinto peanut) also displaying little leaf symptoms [4]. Subsequently, StLL was detected in northern Australia in asymptomatic sugarcane (*S. officinarum* L) [5], in papaya (*Carica papaya* L.) with papaya yellow crinkle disease and in two weed species, *Bonamia pannosa* (R.Br.) Hallier f. and *Indigofera linifolia* (L.f.) Retz [6]. The two weed species both showed proliferation, phyllody and little leaf symptoms with *B. pannosa* showing fasciation as an additional symptom [6]. More recently, StLL was detected in lucerne (*Medicago sativa* L.) with yellowing and little leaf symptoms from the Central West region of New South Wales [7]. In 2019, a StLL-like phytoplasma was detected in Victoria, Australia, in a potato plant (*Solanum tuberosum* L.) with stunted growth and little leaf symptoms. Although the disease incidence was less than 1 % of the potato crop, detection was significant as it was the first report of StLL in potato and in southern Australia. Together, these infections suggest that StLL may have a diverse host range and may be ubiquitous in the Australian environment.

A novel phytoplasma group is generally represented by <97.5 % nucleotide sequence identity of the 16S rRNA gene when compared with described ‘*Ca*. Phytoplasma’ representatives [8]. Phytoplasma strains and species within a ‘*Ca*. Phytoplasma’ 16 Sr group can be assigned a subgroup based on a unique RFLP pattern of the 16S rRNA gene region [8]. Some closely related species, in which the 16S rRNA gene has >97.5 % nucleotide sequence identity, can be delineated based on biological and ecological characteristics such as antibody specificity, geographic location, host range or vector transmission specificity (e.g. ‘*Ca*. Phytoplasma mali’, ‘*Ca*. Phytoplasma pyri’, ‘*Ca*. Phytoplasma prunorum’) [8, 9]. Multilocus sequence analyses (MLSA) subgrouping using phylogenetic analyses of single copy genes that are more variable than the 16S rRNA genes, such as *tuf* (*tu-*elongation factor) [10], *secA* (partial translocation gene) [11], and the *ribosomal protein* (*rp*) gene operon (*rps19-rpl22-rps3*) [12], are often used to provide further support for the assignment of a ‘*Ca*. Phytoplasma’ taxon.

Previous studies showed that StLL in *S. scabra*, sugarcane, papaya and lucerne were very closely related based on RFLP and sequencing of the 16S rRNA gene (generally more than 99 % nucleotide identity) [3–7]. However, StLL was shown to be different (<93 % sequence identity) from other phytoplasmas [3, 5, 7]. Although the previous studies indicate that this phytoplasma is unique, StLL has remained unclassified at the phytoplasma 16 Sr group or subgroup level and is rarely used in phylogenetic analyses. Therefore, in this study, eight StLL strains from the Northern Territory, Queensland and Victoria from various symptomatic host plants (Table 1) were used to characterise this unique phytoplasma as a novel ‘*Ca*. Phytoplasma’ taxon at the molecular level. An MLSA-based approach was used that includes sequences of the 16S rRNA gene, partial *tuf* and *secA* genes and the *rp* gene operon *rps19-rpl22-rps3*.

**Table 1.** The *Stylosanthes* little leaf (StLL) phytoplasma strain used for characterisation and assignment of the taxon, ‘*Candidatus* Phytoplasma stylosanthis’. The representative strain submitted to the Victorian Plant Pathology Herbarium (VPRI) collection in is in bold.

Strain name	Year sampled	Host species	Host symptoms	Origin of strain in Australia
**VPRI 43683**	**2019**	***Solanum tuberosum* L**.	**Little leaf, stunted growth**	**Victoria**
StLL-Bi	2019	*Solanum tuberosum* L.	Little leaf	Victoria
StLL-M7	2019	*Solanum tuberosum* L.	Little leaf	Victoria
StLL-528	1998	*Carica papaya* L.	Yellowing, downward turned petioles, leaf dieback	Northern Territory
StLL-712	1998	*Stylosanthes scabra* Vogel	Little leaf	Queensland
StLL-725	1998	*Stylosanthes scabra* Vogel	Little leaf	Queensland
StLL-778	1998	*Stylosanthes scabra* Vogel	Little leaf	Queensland
StLL-935	1998	*Stylosanthes scabra* Vogel	Little leaf	Queensland

Total DNA was extracted from symptomatic leaf veins and petiole tissue of the three potato plants sampled in 2019 using a modified CTAB and DNeasy Plant Mini Kit (Qiagen) method (Table 1; [13]). DNA concentrations were measured using a NanoDrop spectrophotometer and diluted to approximately 30 ng µl^–1^ for use in PCR. DNA of StLL infected papaya and *Stylosanthes* plants collected in Queensland and the Northern Territory prior to 2019 were provided by the Phytoplasma DNA Collection, initially held at the Charles Darwin University, Darwin but which now reside at the Northern Territory Department of Industry, Tourism and Trade, Darwin, Australia (Table 1). All whole DNA extractions were stored at −20 °C or −80 °C until use.

An approximately 1800 base pair (bp) fragment encompassing the phytoplasma 16S rRNA gene, the 16 Sr/23 Sr intergenic spacer region and part of the 23S rRNA gene was amplified from DNA of the eight StLL strains using a nested PCR [14, 15]. The amplicons were purified and cloned as previously described [16]. Colony PCR, using SP6 and T7 general primers, was used to directly screen ten colonies of each strain for successful ligation of the amplicons [17]. Four cloned amplicons of the expected size for each StLL strain were purified using the QIAquick PCR Purification Kit (Qiagen). The purified amplicons were then Sanger sequenced (Macrogen Korea) in each direction using the SP6 and T7 primers. The sequences from each StLL strain were assembled into consensus sequences using Geneious Prime 2019.2.1 (https://www.geneious.com) and regions with low quality scores were removed. To estimate sequence similarities and undertake phylogenetic comparisons with previously described ‘*Ca*. Phytoplasma’ representatives for classification purposes (Table 2), the sequences were trimmed to a near full length 16S rRNA gene (a 1343 bp region between the R16F2n and m23sr primer sites, equivalent to nucleotide positions 4–1346 in the reference GenBank sequence MT431550). To investigate interoperon sequence heterogeneity of the 16 Sr gene, sequences were trimmed to encompass an approximately 1700 bp region, directly between the R16F2n and m23sr primers [14, 15].

**Table 2.** The ‘*Candidatus* Phytoplasma’ species and strains and associated GenBank accession numbers of the 16S rRNA, *tuf*, *secA* and *rps19-rpl22-rps3* gene sequences used in this study and the pairwise percentage nucleotide sequence identity with ‘*Candidatus* Phytoplasma stylosanthis’ *rrnB* (GenBank accession number: MT431550) are indicated. For simplicity, only the ‘*Candidatus* Phytoplasma stylosanthis’ representative was used for comparisons and is highlighted in bold font. All percentages are derived from the aligned regions used to construct the maximum likelihood trees

Described ‘*Candidatus* Phytoplasma species’	16 Sr group – subgroup*	16S rRNA gene		*Tuf* gene		*secA* gene		*rps19-rpl22-rps3* genes	
		Strain (GenBank No.)	% nucleotide identity	Strain (GenBank No.)	% nucleotide identity	Strain (GenBank No.)	% nucleotide identity	Strain (GenBank No.)	% nucleotide identity
‘*Candidatus* Phytoplasma stylosanthis’ strain:									
‘***Candidatus* Phytoplasma stylosanthis’**	16SrXXXVII-A	*rrnB,* VPRI 43683 (MT431550)	100	VPRI 43683 (MT432813)	100	VPRI 43683 (MT432821)	100	VPRI 43683 (MT461153)	100
		*rrnA,* StLL-Bi (MT431551)	100						
Previously described ‘*Candidatus* Phytoplasma’ representatives:									
‘*Ca*. Phytoplasma asteris’	16SrI-B	OAY (M30790)	91.01	AY-1 (JQ824205)	68.27	Cph (EU168722)	67.90	AVUT (AY264855)	57.18
‘*Ca*. Phytoplasma aurantifolia’	16SrII-B	WBDL (U15442)	89.78	WBDL (JQ824276)	69.29	WBDL (EU168731)	79.01	LWB (EF186815)	61.22
‘*Ca*. Phytoplasma australasia’	16SrII-D	PpM (Y10096)	89.91	TBB-KG (JQ824250)	70.81	TBB (EU168728)	80.86	TBB (EF193373)	60.48
‘*Ca*. Phytoplasma pruni’	16SrIII-A	*rrnA*, PX11CT1 (JQ044393)	93.75	CX (JQ824211)	72.59	WX_California (KJ462026)	69.14	PX11CT1 (JQ360958)	62.81
		*rrnB*, PX11CT1 (JQ044392)	93.76						
‘*Ca*. Phytoplasma ulmi’	16SrV-A	EY1 (AY197655)	96.28	EY1 (JQ824225)	73.86	EY1 (KJ462034)	85.80	EY1 (JN851864)	72.57
‘*Ca*. Phytoplasma ziziphi’	16SrV-B	JWB-G1 (AB052876)	96.11	JWB 19/2008 (JQ824203)	74.37	JWB (KJ462036)	83.33	JWB (AY197681)	72.93
‘*Ca*. Phytoplasma rubi’	16SrV-E	RuS (AY197648)	96.13	RuS (JQ824210)	74.62	RuS (KJ462043)	85.19	RuS (AY197668)	74.02
‘*Ca*. Phytoplasma balanitae’	16SrV	BltWB, (AB689678)	95.24	na	–	BeWB1 (MH816938)	84.57	BltWB (AB689679)	72.85
‘*Ca*. Phytoplasma trifolii’	16SrVI-A	CP (AY390261)	97.32	CP-1 (JQ824231)	77.92	PWB (EU168742)	82.72	CP (EF183486)	73.71
‘*Ca*. Phytoplasma sudamericanum’	16SrVI-I	PassWB-Br3 (GU292081)	95.98	na	–	na	–	na	–
‘*Ca*. Phytoplasma fraxini’	16SrVII-A	AshY1 (AF092209)	96.58	ASHY3 (JQ824263)	80.46	ASHY-1 (EU168745)	82.72	ASHY-1 (EF183492)	73.71
‘*Ca*. Phytoplasma luffae’	16SrVIII-A	*rrnA*, LfWBR (AF248956)	97.25	LfWBR (AF086617)	86.04	na	–	na	–
		*rrnB*, LfWBR (AF353090)	97.25						
‘*Ca*. Phytoplasma phoenicium’	16SrIX-D	A4 (AF515636)	93.68	PEY (JQ824256)	69.04	PPWB (EU168746)	83.33	PEY (EF186802)	66.75
‘*Ca*. Phytoplasma mali’	16SrX-A	AP15 (AJ542541)	91.36	AP-15 (JQ824216)	71.57	AP-15 (EU168747)	80.86	AP15 (EF193366)	58.32
‘*Ca*. Phytoplasma pyri’	16 SrX-C	PD1 (AJ542543)	91.59	PD (JQ824247)	72.34	na	–	PD (EF193370)	59.34
‘*Ca*. Phytoplasma spartii’	16SrX-D	SpaWB (X92869)	90.63	Si04-S4 (FR686504)	74.87	na	–	na	–
‘*Ca*. Phytoplasma prunorum’	16 SrX-F	ESFY-G1 (AJ542544)	91.66	GSFY1 (EU103617)	71.32	GSFY1 (EU168748)	81.48	na	–
‘*Ca*. Phytoplasma oryzae’	16SrXI-A	RYD-J (D12581)	94.49	NGS (JQ824288)	70.28	NGS (EU168750)	78.40	na	–
‘*Ca*. Phytoplasma cirsii’	16SrXI-D	CirYS (KR869146)	94.71	na	–	CirYS (KU557489)	79.63	na	–
‘*Ca*. Phytoplasma solani’	16SrXII-A	STOL11 (AF248959)	89.23	STOL11 (JQ797670)	69.29	STOL (EU168752)	72.22	P-TV (EF193364)	56.92
‘*Ca*. Phytoplasma australiense’	16SrXII-B	AUSGY (L76865)	90.25	AUSGY (JQ824254)	69.04	AGY (KJ462054)	74.15	PYCL (AY303560)	57.33
‘*Ca*. Phytoplasma japonicum’	16SrXII-D	JHP (AB010425)	89.82	na	–	na	–	HYDP (AY264868)	57.11
‘*Ca*. Phytoplasma fragariae’	16SrXII-E	StrawY (DQ086423)	89.68	YN-169 (KJ144900)	69.04	CPF (EU168751)	72.22	YN-169 (EU338446)	58.16
‘*Ca*. Phytoplasma convolvuli’	16SrXII-H	BY-S57/11 (JN833705)	86.96	Ud12_272 (KJ469710)	70.81	na	–	na	–
‘*Ca*. Phytoplasma hispanicum’	16SrXIII-A	MPV (AF248960)	89.67	na	–	MPV (EU168753)	72.84	MPV (EF193365)	56.64
‘*Ca*. Phytoplasma meliae’	16SrXIII-G	ChTY-Mo3 (KU850940)	89.83	na	–	ChTYXIII-Mo3 (KU850948)	74.69	ChTYXIII-Mo3 (KU850944)	57.13
‘*Ca*. Phytoplasma cynodontis’	16SrXIV-A	BGWL-C1 (AJ550984)	94.86	na	–	WH04 (KY495289)	77.02	na	–
‘*Ca*. Phytoplasma brasiliense’	16SrXV-A	HibWB26 (AF147708)	89.84	SUV (JQ824234)	69.54	na	–	na	–
‘*Ca*. Phytoplasma graminis’	16SrXVI-A	SCYLP (AY725228)	86.55	na	–	na	–	na	–
‘*Ca*. Phytoplasma caricae’	16SrXVII-A	PAY (AY725234)	86.04	na	–	na	–	na	–
‘*Ca*. Phytoplasma americanum’	16SrXVIII-A	APPTW12-NE (DQ174122)	90.05	SRL1-PA (MN227135)	69.67	SRL1-PA (MN227136)	75.93	APPTW10.NE (EF193362)	68.35
‘*Ca*. Phytoplasma castaneae’	16SrXIX-A	CnWB (AB054986)	93.75	na	–	na	–	na	–
‘*Ca*. Phytoplasma rhamni’	16SrXX-A	BAWB (X76431)	91.00	RhCa (JQ824207)	70.69	na	–	BWB-bis (KF498659)	64.80
‘*Ca*. Phytoplasma pini’	16SrXXI-A	Pin127S (AJ632155)	94.87	na	–	MDPP (KU242429)	76.54	na	–
‘*Ca*. Phytoplasma palmicola’	16SrXXII-A	LYDM-178^R^ (KF751387)	93.38	LYSS (EU413952.1)	72.08	MZ13-012 (LR029127)	78.40	na	–
‘*Ca*. Phytoplasma allocasuarinae’	16SrXXXIII-A	alloY, (AY135523)	90.13	na	–	na	–	na	–
‘*Ca*. Phytoplasma lycopersici’	16SrI-Y	THP (EF199549)	88.62	na	–	na	–	na	–
‘*Ca*. Phytoplasma omanense’	16SrXXIX-A	IM-1 (EF666051)	93.76	na	–	na	–	na	–
‘*Ca*. Phytoplasma tamaricis’	16SrXXX-A	SCWB1 (FJ432664)	90.92	na	–	na	–	na	–
‘*Ca*. Phytoplasma costaricanum’	16SrXXXI-A	SoySTc1 (HQ225630)	90.63	na	–	na	–	na	–
‘*Ca*. Phytoplasma malaysianum’	16SrXXXII-A	MaPV (EU371934)	97.10	na	–	MaPV (FJ755005)	84.57	na	–
‘*Ca*. Phytoplasma wodyetiae’	16SrXXXVI-A	Bangi-2 (KC844879)	93.74	na	–	na	–	na	–
‘*Ca*. Phytoplasma noviguineense’	nd	BCS-Bo (LC228755)	94.50	na	–	na	–	BCS-Bo (LC228762)	67.00
Provisionally proposed species [8]:									
‘*Ca*. Phytoplasma cocostanzaniae’	16SrIV	LD (X80117)	94.94	na	–	TLD Chambezi (KJ462072)	77.78	na	–
‘*Ca*. Phytoplasma palmae’	16SrIV	LY (U18747)	95.09	na	–	LYSS (EU267187)	79.01	LY (EF193382)	66.56
‘*Ca*. Phytoplasma vitis’	16SrV	FD70 (AF176319)	96.21	FD-D Veneto 107/06 (JQ824294)	74.62	FD-D Veneto 107/06 (KJ462042)	85.19	FD70 (AY197663)	73.85
Buckland valley grapevine yellows	16SrXXIII	BVGY (AY083605)	87.62	BVGY (Constable *et al.* unpublished)	69.04	BVGY (Constable *et al.* unpublished)	72.22	BVGY (Constable *et al.* unpublished)	56.01

*Group indicated by roman numeral; sub-group designated by a letter.

ND: Not Determined; NA: Not Available

## Novel ‘*Candidatus* Phytoplasma’ taxon

The 16S rRNA gene sequences were aligned using MAFFT [18]. Pairwise sequence similarities between all StLL 16S rRNA gene sequences, including the original sequences deposited on the NCBI database (GenBank accession numbers: Y17055 and AJ289192), showed 99.93–100 % nucleotide sequence identity (Table S1a, available in the online version of this article). The StLL 16S rRNA gene sequences (GenBank accession numbers: Y17055, AJ289192, MT431550-MT431557) had less than 97.5 % nucleotide sequence identity with other ‘*Ca.* Phytoplasma’ representatives. The most closely related phytoplasmas species were ‘*Ca.* Phytoplasma luffae’ in group 16SrVIII-A and ‘*Ca.* Phytoplasma trifolii’ in group 16SrVI-A with which the eight StLL strains shared 97.17–97.25 % and 97.25–97.32 % sequence identity, respectively.

**Fig. 1.**
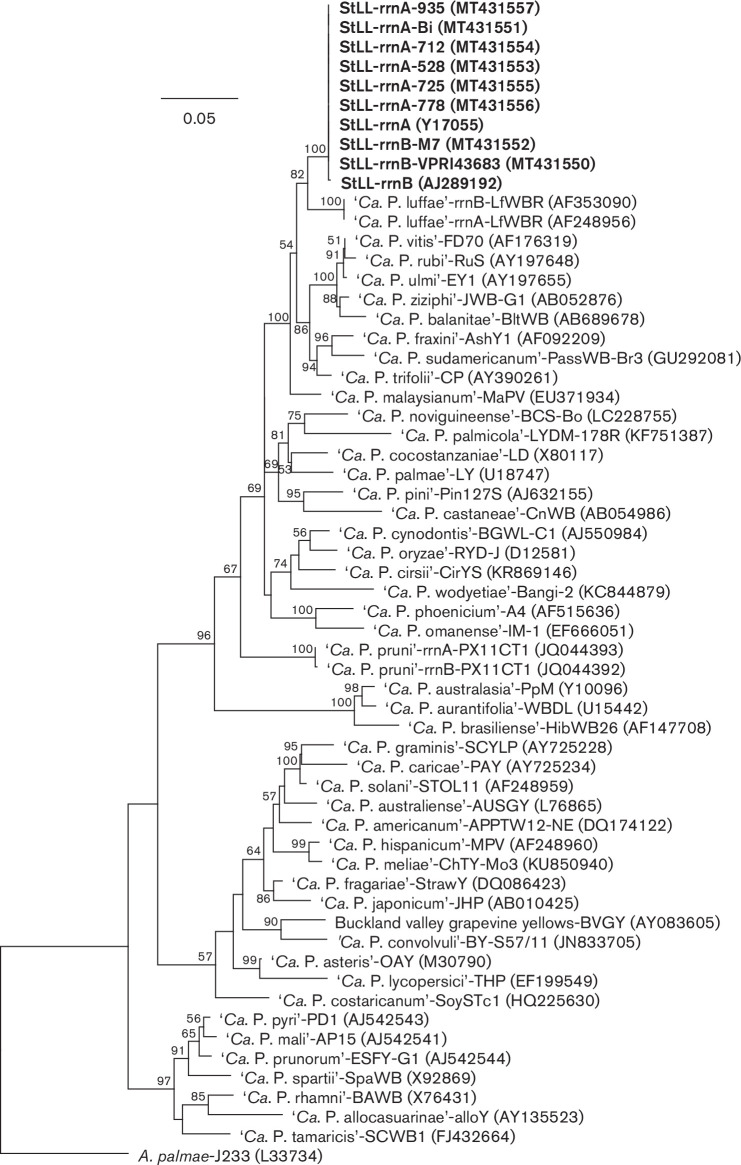
Phylogenetic tree inferred from analyses of 16S rRNA gene sequences of eight *Stylosanthes* little leaf (StLL) phytoplasma strains (highlighted in bold font) and previously described ‘*Candidatus* Phytoplasma’ reference strains. *Acholeplasma palmae* served as the out group. Maximum likelihood analyses were done using RaxML implemented in Geneious Prime 2019.2.1. Numbers at branch nodes indicate the percentage of bootstrap support associated with clustered taxa, and only support percentages of 50 % and above are shown. Branch lengths indicate the number of nucleotide substitutions per site (see bar).

To infer the phylogenetic position of StLL strains within the ‘*Ca.* Phytoplasma’ genus, a maximum likelihood tree was constructed in RAxML version 4 [19] through the Geneious Prime 2019.2.1 plug-in with the 16S rRNA gene sequence alignments. The bootstrap method with 1000 replicates was used to test the reliability of estimated tree topologies. The results suggest that ‘*Ca*. Phytoplasma luffae’ and StLL phytoplasmas share a common ancestor, with 82 % bootstrap support for this relationship (Fig. 1), and ‘*Ca.* Phytoplasma luffae’ and StLL form a cluster with other phytoplasma representatives in the groups 16SrV, 16SrVI, 16SrVII, and 16SrXXXII (Fig. 1).

**Fig. 2.**
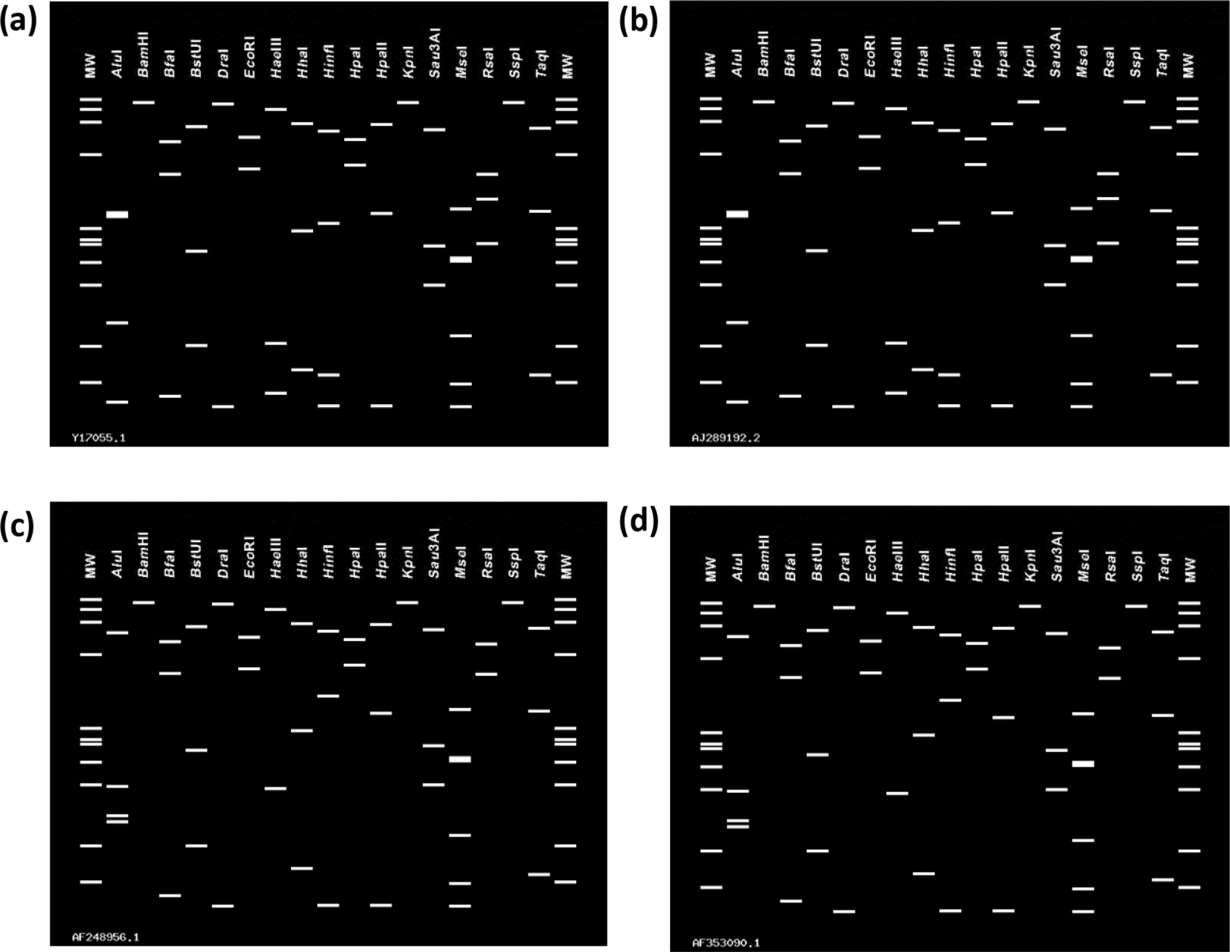
Comparison of *in silico* RFLP patterns obtained with *i*PhyClassifier from the digestion of the 1245 base pair 16S gene fragments of *Stylosanthes* little leaf (StLL) phytoplasma and ‘*Candidatus* Phytoplasma luffae’ using 17 enzymes for phytoplasma classification. (a) StLL phytoplasma *rrnA* gene (GenBank no. Y17055), (b) StLL phytoplasma *rrnB* gene (GenBank no. AJ289192), (c) ‘*Candidatus* Phytoplasma luffae’-LfWBR *rrnA* gene (GenBank no. AF248956), and (d) ‘*Candidatus* Phytoplasma luffae’-LfWBR *rrnB* gene (GenBank no. AF353090).

Virtual RFLP analysis of the StLL 16S rRNA gene sequences using *i*PhyClassifier [20] indicated a unique pattern that was most similar to *‘Ca.* Phytoplasma luffae’ [21] (16SrVIII-A; Fig. 2), with a similarity coefficient of 0.83. Since the similarity coefficient is below the demarcation threshold of 0.85 [20], the StLL phytoplasma may represent a new 16 Sr group. To confirm the virtual RFLP (Fig. 2) *in vitro*, a 1247 bp 16 Sr PCR amplicon that does not include the heterogeneous region was amplified directly from the StLL-VPRI 43683 DNA extract in a nested PCR using the R16F2n/R16R2 PCR primers. The resultant R16F2n/R16R2 PCR amplicons were digested with the restriction enzymes *Alu*I, *Bfa*I, *BstU*I, *Hae*III, *Hha*I, *Mse*I, *Rsa*I, and *Sau3*AI in separate reactions as per the manufacturer protocols. These nine *in vitro* RFLP profiles supported those generated by *i*PhyClassifier (data not shown).

The first 16S *rrnB* gene sequence of StLL phytoplasma from a detection in *Stylosanthes* (GenBank accession number: AJ289192) and the most recent strain in potato (*rrnB*, GenBank accession number MT431550) are 99.93 % similar in sequence over the 1343 bp region between the R16F2n and m23sr primer sites. Both phytoplasma strains have less than 97.5 % nucleotide sequence identity to the same fragments of the 16S rRNA genes from all other described ‘*Ca.* Phytoplasma’ taxa. Therefore, according to the International Organization for Mycoplasmology (IRPCM) recommendations, StLL can be assigned to a novel, distinct ‘*Ca.* Phytoplasma’ taxon. This is further supported by the unique RFLP pattern of the StLL 16S rRNA gene and its unique geographic origin of Australia. It is proposed that StLL is designated the representative of a novel ‘*Candidatus*’ taxon, ‘*Candidatus* Phytoplasma stylosanthis’. The results also indicate that ‘*Ca.* Phytoplasma stylosanthis’ forms a unique 16Sr group, which could be designated as 16SrXXXVII. As the first known member in the unique 16Sr group, ‘*Ca*. Phytoplasma stylosanthis’ belongs to the 16SrXXXVII-A subgroup.

## Description of ‘*Candidatus* Phytoplasma stylosanthis’

‘*Candidatus* Phytoplasma stylosanthis’ (sty.los.an′this. N.L. gen. n. stylosanthis of *Stylosanthes*, referring to a plant genus in which the phytoplasma was discovered).

The reference strain is from the most recent detection in a potato plant in Victoria, Australia, which had little leaf symptoms and stunted growth. A sample of potato tissue with ‘*Ca.* Phytoplasma stylosanthis’ (GenBank accession nos.: MT431550, 16S rRNA gene; MT432821, partial *secA* gene; MT432813, partial *tuf* gene; MT461153, partial *rps19-rpl22-rps3* operon) has been deposited with the Victorian Plant Pathology Herbarium (herbarium ref no: VPRI 43683) at Agriculture Victoria – Bundoora. The 16S rRNA gene sequence of the reference strain (VPRI 43683) is 99.94 % similar to the R16F2n/m23sr region of the original *rrnB* sequence described from *Stylosanthes scabra* in 2001 [3], which also had little leaf symptoms (GenBank accession no.: AJ289192). Additional gene regions were not available for the original StLL strains described in 1999 [4] and 2001 [3], thus further sequence analyses with these strains were not possible.

‘*Candidatus* Phytoplasma stylosanthis’ [(Mollicutes) NC; na; O, wall-less (GenBank accession number MT431550); Oligonucleotide sequences of unique regions within the 16S rRNA gene are: 5′ – TTTAACAAAGG – 3′ (41–51 bp), 5′ – GCTAGCTAGAGTG – 3′ (474–486 bp), 5′ – TTGTCGTTAGTTACCAGCACGTTATGGTGGGGACTTTAGCGAGACTGCCAATT-AAAA – 3′ (934–990 bp), 5′ – CGCTGAAACGTGAGTTTTTGGCTAATCTCAAAAAAGC – 3′ (1077–1113 bp), 5′ – TTGACAATACCCGAAAGCAGTGACTTAACTTCGCAAGAAGAGGGAACTGTCTAAGGTAGGGTT– 3′ (1241–1303 bp)].

## Interoperon sequence heterogeneity in the 16s-23s rRNA spacer

The intergenic region encoding the tRNA^Ile^ gene (100 bp) was not recovered for some cloned PCR fragments of the eight ‘*Ca.* Phytoplasma stylosanthis’ strains used in this study. This observation supports previous findings of interoperon sequence heterogeneity. The original ‘*Ca.* Phytoplasma stylosanthis’ 16S rRNA gene sequence that lacks the tRNA^Ile^ gene was designated as *rrnA*, and the second 16S rRNA gene sequence encoding the tRNA^Ile^ gene as *rrnB* [3]. Despite the interoperon heterogeneity, the *rrnA* and *rrnB* sequences within each StLL phytoplasma had high (>99.6 %) nucleotide identity when the tRNA^Ile^ gene was excluded, which has been shown previously [3]. Similar 16S rRNA interoperon sequence heterogeneity also occurs in ‘*Ca.* Phytoplasma luffae’ [21], representing an additional character shared between StLL and this species, and may indicate that they share a common ancestor. However, where the intergenic region was present, the region of ‘*Ca.* Phytoplasma luffae’ (63 bp) is smaller than that of ‘*Ca.* Phytoplasma stylosanthis’ (102 bp) and they only share 79 % nucleotide identity.

## Additional features

An MLSA-based approach was used to further define ‘*Ca.* Phytoplasma stylosanthis’ and investigate the phylogenetic relationships with other ‘*Ca.* Phytoplasma’ taxa. MLSA was done using partial sequences of the *tuf* (GenBank accession numbers: MT432806-MT432813) [10] and *secA* (GenBank accession numbers: MT432814-MT432821) [11] genes, as well as the combined sequence of the partial S19 (*rps19*), complete L22 (*rpl22*) and partial S3 (*rps3*) genes (GenBank accession numbers: MT461146-MT461153) [12] for all eight ‘*Ca.* Phytoplasma stylosanthis’ strains. PCR amplicons of the expected sizes from each strain were purified, cloned and processed for forward and reverse Sanger sequencing as described for the 16S rRNA gene. The consensus sequence for each gene region of each strain was confirmed to be of phytoplasma origin by blastn analyses [22]. The sequences were aligned with each other and with previously described phytoplasma representatives using MAFFT [18] to compare the nucleotide sequence similarity and to estimate the phylogeny of the genes. All eight ‘*Ca.* Phytoplasma stylosanthis’ strains shared a high nucleotide sequence identity among the genes that were analysed, regardless of the year, host, or region from which they were collected (Table 1 and S1b–d). Indeed, the *tuf* and *secA* gene regions were identical among all eight strains, while their *rps19-rpl22-rps3* gene operon shared between 99.7 and 100 % nucleotide sequence similarity. Differences in this operon included two transition mutations (positions 64 and 1237) and one transversion mutation (position 718) in StLL-Bi, a deletion in StLL-725 at position 828, and an insert (T) was identified at position 881 for strains StLL-M7 and StLL-935.

**Fig. 3.**
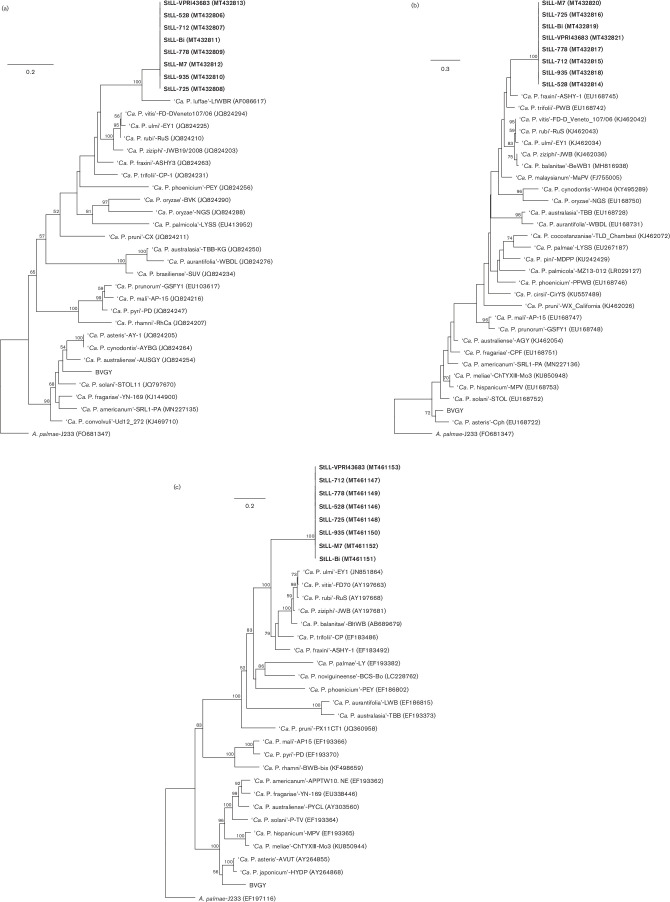
Phylogenetic trees of eight *Stylosanthes* little leaf (StLL) phytoplasma strains and other ‘*Candidatus* Phytoplasma’ taxa inferred from analyses of the phytoplasma gene regions (a) *tuf*, (b) *secA*, and (c) *rps19-rpl22-rps3*. Maximum likelihood analyses were done using RaxML implemented in Geneious Prime 2019.2.1. Orthologous genes from *Acholeplasma palmae* were used as the out group to root each tree. Numbers at branch nodes indicate the percentage of bootstrap support associated with clustered taxa, with support percentages of 50 % and above shown. Branch lengths indicate the number of nucleotide substitutions per site (see bar).

Similar to the results of the 16S rRNA gene, the *tuf* gene of ‘*Ca.* Phytoplasma stylosanthis’ had one of the highest nucleotide sequence identities (86.0 %) with ‘*Ca.* Phytoplasma luffae’ (16SrVIII-A) (Table 2) and they clustered together in the maximum likelihood tree (100 % bootstrap support for this relationship; Fig. 3a), also suggesting that they share a recent common ancestor. The nucleotide sequence identity and the maximum likelihood gene trees of the *secA* and *rps19-rpl22-rps3* genes consistently suggested a close relationship of StLL with members of groups 16SrV, 16SrVI, and 16SrVII (Table 2, Fig. 3b, c). The *secA* and *rps19-rpl22-rps3* gene regions of ‘*Ca.* Phytoplasma stylosanthis’ strains shared 82.7–85.8 % and 73.6–74.0 % nucleotide sequence identity, respectively, with the members in these three closely related phytoplasma groups (Table 2). In the *secA* gene tree, the cluster also included ‘*Ca.* Phytoplasma malaysianum’ (16SrXXXII-A, Fig. 3b), which was also observed in the 16 Sr tree. There were no *secA* or *rps19-rpl22-rps3* gene sequences of ‘*Ca.* Phytoplasma luffae’ available for comparison with StLL (Table 2). As the sequences of different gene regions are not consistently available for all phytoplasma species, it is challenging to compare the relationships between the phytoplasma groups using different gene regions. Nevertheless, these results also support the assignment of the novel ‘*Candidatus*’ taxon, ‘*Candidatus* Phytoplasma stylosanthis’.

## Supplementary Data

Supplementary material 1
